# The enablers, barriers and preferences of accessing radiation therapy facilities in the rural developed world – a systematic review

**DOI:** 10.1186/s12885-017-3790-7

**Published:** 2017-11-28

**Authors:** Sandra C. Thompson, Shelley Cheetham, Siddhartha Baxi

**Affiliations:** 1 Western Australian Centre for Rural Health, The University of Western Australia, 35 Stirling Highway, Perth, WA 6009 Australia; 2 Aboriginal and Rural Health Care, The University of Western Australia, 35 Stirling Highway, Perth, WA 6009 Australia; 3 South West Radiation Oncology Service, South West Health Campus, Corner of Bussell Hwy & Robertson Drive, Bunbury, WA 6230 Australia

**Keywords:** Utilisation, Radiation therapy, Radiation therapy, Regional, Rural, Barriers, Enablers, Access, Decision making

## Abstract

**Background:**

Utilisation of radiation therapy for regional Australia and around the world has been the focus of much health policy the last decade. Radiation therapy centres have been built in Australian regional and rural areas to improve access to radiation therapy and reduce the tyranny of distance as a barrier to access. After this the enablers, barriers and perceptions of patients has been evaluated to determine utilisation once centres have been built. Thisreview looks the impact of rural radiation services in the developed world, barriers and enablers of establishing a rural radiation centre, and patients’ and service providers’ perspectives and preferences around the uptake of rural radiation therapy.

**Methods:**

Online search of peer reviewed literature was undertaken using MeSH terms relating to the topic. Inclusion criteria were regional radiation therapy centres in developing countries, any year of publication, in English, and qualitative or quantitative methodologies. Articles were reviewed by two authors with conflicts discussed with a third.

**Results:**

Twenty three studies addressed the theme directly. Distance barriers have been overcome by building regional centres and health economic burden was lower for government service providers with this strategy. However distance still plays an important role in influencing uptake of radiation therapy. Cultural expectations, influence of the family doctor and perception of care was influential. Carer support, duration of displacement from home, financial impact of the required care and seasonal weather were practical factors on a patient’s decision.

**Conclusions:**

Regional radiation therapy centres have improved access to radiation therapy in developing countries. However the complex nuances between socio-economic, cultural and health system factors that influence regional patient’s decision making bears further consideration, as distance is not the only issue.

## Summary points

- Regional and rural radiation therapy centres have improved access by breaking down the barrier of distance.
- Looking beyond the barrier of distance to improve access to radiation therapy, the socio-economic and cultural factors that influence patient decisions need to be appreciated.
- The information on this topic is lacking for why the nearest regional radiation therapy centres are not always utilised by patients.

This is best done with government and consumer input prospectively to ensure productive knowledge translation in establishing radiation therapy centres.

## Background

The development of regional radiation therapy centres in Australia has been a significant area of attention and development for our health system in recent times. The first regional radiation therapy centre to explore the feasibility of small or single machine units to operate effectively in regional areas was opened in the mid-2000s, in regional New South Wales. Since then, 19 regional and rural centres have been established in Australia [1]. In response to compelling data that indicated that distance is an important contributory barrier to the access and utilisation of radiation therapy services [2, 3] and impacts on survival [4]. This illustrates that regional centres may be one solution to overcoming the barrier of distance. Despite progress in distributing radiation therapy outside of major urban centres, utilisation of radiation therapy remains below the 49% predicted when all cancer cases in Australia are considered [5]. The utilisation rate in rural and regional Australia is 19% compared to 36% in metropolitan areas [6]. This shows that utilisation and access to radiation therapy, whether it is due to patient or health system factors, still remains a challenge despite the establishment of regional centres. While this appears to be improving over time [7], there is a need to better understand how radiation therapy utilisation and access can be improved. The objective of this review is to explore published literature around the impact of rural radiation therapy services in developed countries.

This literature review investigates three key issues:

1. Utilisation and the impact of rural radiation services in the developed world
2. Barriers and enablers of establishing a rural radiation centre
3. Patients’ and service providers’ perspectives and preferences, and the enablers and barriers around the uptake of rural radiation therapy

The rationale was to understand what the establishment of regional centres has achieved, what barriers remain, and the perspectives of patients on accessing radiation therapy closer to home.

## Methods

An electronic online search of peer reviewed literature was undertaken using MeSH terms related to “regional/remote +/− cancer/oncology/radiation therapy/radiation oncology + facility/service/hospital/centre/center (& plural terms). Keywords used in the search included combinations of rural/regional/remote, radiation therapy/radiation oncology, patient satisfaction/preferences and health service quality/access/evaluation. A total of 453 papers were retrieved by this search. The following inclusion criteria used to identify relevant papers:

* Services related to rural/regional/remote facilities and/or
* Services related to radiation therapy service provision and/or
* Any year of publication and
* Any developed country, for example; Canada/United States of America/United Kingdom/Australia/New Zealand/Europe
* Published in English language

Quantitative and qualitative methodologies and health economic analyses were all considered within the scope of this review.

**Fig. 1:**
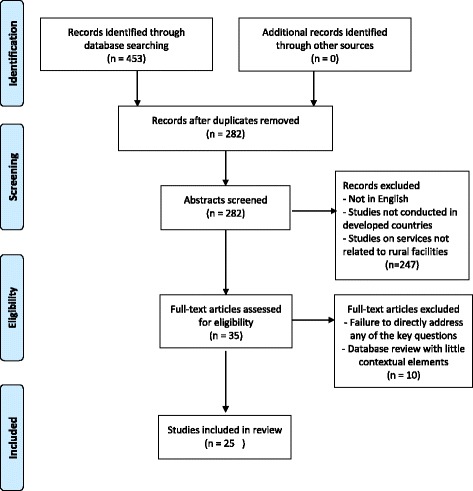
PRISMA Flow Diagram

Studies were excluded if they reported on a metropolitan centre, were not related to radiation therapy service provision, or reported on initiatives in a developing country. The process of reviewing the literature was undertaken by two authors; where there were discrepant views around the relevance, additional authors were used to assess and resolve issues through consensus to ensure adherence to the inclusion criteria. This is described in the Preferred Reporting Item for Systemic Reviews (PRISMA) Fig. 1.

## Results

### Quality and type of data

Table 1 summarises the 25 relevant publications that met the inclusion criteria and included in this review. Publication year ranges from 1996 to 2016. The majority of the studies originated from North America (*n* = 15) and Australia (*n* = 9), with one study from the United Kingdom. Most of studies described issues around a specific cancer type (breast (*n* = 10), colorectal (*n* = 2), prostate (*n* = 1)) while 12 studies covered more than one cancer type. Most studies (*n* = 20) employed a quantitative methodology, while only 5 utilised a qualitative approach. The sample size (*n* = 18) for the majority of studies was >1000 patients with only four having a sample size less than 1000 patients, and 3 not describing sample size (generally described catchment area by number of hospitals from which data was gathered). Given that the majority of the studies were quantitative with a large sample size, 14 presented multivariate analytical data with the endpoints measured listed in Table 1. Clinical, treatment utilisation, health economic and distance endpoints were commonly described. Qualitative reports included factors influencing patient decisions around financial, awareness of availability of services and the option of radiation therapy, and education issues.

**Table 1:** Studies included in the analysis

Research question:			Location	Author	Sample size	Cancer type	Study years	Review type	Endpoint/Question
1	2	3							
Y	N	N	Australia	Baade	6848	CRC^c^	1996–2006	Population based	Disease free survival
Y	N	N	Scotland	Campbell	1314	CRC/Lung	1995–1996	Historical cohort	Time between referral and treatment
Y	N	N	USA	Dragun	11,914	Breast	1998–2007	Retrospective chart	Radiotherapy utilisation after breast surgery
Y	N	N	Canada	French	Unclear	Any	1986–1999	Cancer registry	Utilization of radiotherapy by geography
Y	N	N	Canada	Lengoc	1001^a^	Any		Population based	Factors influencing referral patterns from family doctors
Y	N	N	USA	Martinez	7509	Breast	2000–2006	Database	Radiotherapy utilisation in rural versus metro regions
Y	N	N	USA	Schroen	11,597	Breast	1996–2000	Cancer registry	Utilisation by distance to radiotherapy facility
Y	N	Y	Canada	Billar	180,219	Breast	2000–2009	Database	Radiotherapy utilisation for early stage breast cancer
Y	N	Y	Australia	Clavarino	47	Any	1999–2000	Cross sectional	Impact of traveling on cancer patients and their carers
Y	N	Y	USA	Freeman	5541	Breast	1998–2007	Retrospective chart	Surgical and radiotherapy choices for breast cancer patients
Y	N	Y	Australia	Hegney	17	Any	2001	Population based	Burden of travel/accomodation/finances/psychology on ones care
Y	N	Y	Australia	Roder	30,299	Breast	1998–2010	Database	Factors influencing treatment decisions for breast cancer in remote Australia
Y	N	Y	USA	Schootman	6988	Breast	1991–1996	Database	Breast radiotherapy utilisation for rural women
Y	N	Y	Australia	Sharma	1423	Breast/prostate	2010–2013	Database	Influenc of change in distance by opening a radiotherapy facility
Y	N	Y	Australia	Zucca	1453	8 commonest		Population based	Travel burden and financial difficulties on treatment decisions
Y	Y	N	USA	Baldwin	27,143	CRC	1992–1996	Database	How distance to facility influences outcome
Y	Y	N	Australia	Barton	26,081	Any	1996–1998	Population based	Radiotherapy utilisation in NSW
Y	Y	Y	USA	Cetnar	1169	Prostate	2004–2009	Patterns of Care	How geography influences patterns of prostate radiotherapy utilisation
Y	Y	N	Canada	Roberts	8000	Any	2002	Cross sectional	Economic influences for the delivery of radiotherapy
Y	Y	N	Canada	Tyldesley	90,358	Breast/lung/prostate	1997–2007	Cancer registry	Cancer incidence versus radiotherapy utilisation in rural areas
Y	Y	N	Australia	Underhill	161^b^	Any	2005	Cross sectional	Influence of distance to radiotherapy service in regional Australia
N	Y	N	USA	Stafford	266	Breast	1990–1992	Population based	Influence of education of specialists and patients on treatment options
N	N	Y	Australia	Henry	1778	Any	2009	Retrospective chart	Radiotherapy utilisation variation by distance from radiotherapy centre
N	N	Y	USA	Wheeler	1938	Breast	2003–2005	Database	Influence of distance on treatment decisions
N	N	Y	USA	Celaya	2861	Breast	1998–2001	Database	Influence of distance to facility and season on treatment decisions

Research Question 1: What is the known impact of rural radiation services in the developed world?

Research Question 2: What is known about the barriers and enablers of establishing a rural radiation center?

Research Question 3: What is known about the patient’s preferences, perspective, enablers and barriers around uptake of rural radiotherapy?

^a^1001 surveys sent

^b^161 regional hospitals

^c^Colo-rectal cancer

#### What is the known about access and utilisation, and the impact of rural radiation services in developed countries?

Of the 26 included studies, 21 addressed the questions of access and utilisation issues and the impact of rural radiation services in developed countries. A high level view of resourcing was provided by Underhill et al. who surveyed 161 Australia rural hospitals to illustrate that only 6% had an oncology unit and 7% had a dedicated surgical oncologist [8].

Several studies discussed the detrimental impact of distance on outcomes. Baade described that in Queensland, for patients with colorectal cancer, for every 100 km away from a radiation therapy centre there was a 6% relative detriment in overall survival [4]. Roder et al. described that patients in regional areas were less likely to have breast conserving therapy and were influenced by the issue of access to a radiation therapy centre as well as the case load of the surgeon [9]. Zucca examined data which showed that those living in outer regional and remote areas had the greatest travel burden, with 61% travelling at least 2 h one way and 49% having to live away from home to receive treatment. This group experienced greater financial difficulties [10]. Few Australian studies described the impact of the establishment of regional radiation therapy services on outcomes or utilisation. Barton identified an improvement in radiation therapy utilisation rates from 30% in 1991 to 38% in 1998, attributed in part to the establishment of these centres [7]. This was echoed by Sharma and colleagues who supported the view that establishing satellite centres to treat the more common cancers in less urban environment had improved access for patients (ref).

Data from other developed countries described a similar challenge and impact. Baldwin et al. (USA) reported the average distance travelled by a rural patient was 49 miles (75 km), and noted that 20–25% of patients bypass their nearest centre [11]. A Canadian report identified there was a relatively stable but continued under-utilisation of breast radiation therapy as part of a breast conservation protocol between 1998 and 2000 which required an increased utilisation of partial breast irradiation schedules [12]. Distance remained a factor affecting uptake according to Tyldesley et al. in the states of Ontario and British Columbia respectively [13, 14]. A similar finding was reported by Martinez et al. who found that rural patients with breast cancer were less likely to undergo breast radiation therapy [15]. Schroen et al. found a similar trend in the state of Virginia, United States [3]. European data is limited, however Campbell and colleagues observed that rurality had a minor impact on time to receiving treatment in Scotland, but that rural patients were less likely to receive radiation therapy for colorectal carcinoma when indicated [16].

Data around rurality and cultural sub-populations in different countries were analysed in a number of studies. In sequential publications analysing the same cohort of patients, Dragun et al. and Freeman et al. demonstrated that there was underutilisation of breast conserving therapy (which consists of surgery and radiation therapy as a combination treatment) in Kentucky, United States. The Appalachian women of the region were most likely to favour breast conserving surgery despite not accepting breast radiation therapy, thereby compromising their optimal outcomes. Distance and access from treatment were factors in this patient group [17, 18].

A novel question asked by Lengoc and colleagues was regarding the knowledge awareness and involvement of family physicians in British Columbia, Canada around utilisation of palliative radiation therapy for breast cancer care. They noted that rural family physicians were more involved in palliative care and metastatic breast cancer management than metropolitan family physicians, and that their awareness of the state’s radiation therapy facilities was equivalent to metropolitan family physicians [19].

The question of health economics when comparing centralised metropolitan versus de-centralised rural services was explored by Roberts and colleagues who identified that in the Canadian environment, centralised radiation therapy services posed a greater economic burden to the health system and patient [20].

#### What is known about the barriers and enablers of establishing a rural radiation centre?

Knowledge on the barriers and enablers of establishing rural radiation therapy services revolve around health system and community stakeholder issues. While it was expected that these are complex interactions which relate to the political and socio-economic context of the country in question, academic publications exploring these issues were limited. Baldwin et al. made the point that 19.4% to 26% of patients by-passed their nearest radiation therapy service suggesting that some patients may perceive a trade-off between travel and quality care [11] and providing insight into better understanding the decision making process from the patient’s perspective. Barton et al. published data that suggested a rural oncology service without a radiation therapy service still has a major impact on improving utilisation rates [7].

#### What is known about the patient’s perspective, enablers and barriers around uptake of rural radiation therapy?

Several studies explored patient’s reasoning for a decision to have treatment and where to have treatment. Roder et al. provided grounding findings in the Australian context that rural patients were less likely to have breast conserving surgery, and if they had breast conserving surgery, they were less likely to have adjuvant radiation therapy [9]. Clavarino et al. and Hegney et al. made the comment that carers and their children needed significant help to support patients they were caring for to undergo treatment to such an extent that the unmet need for carers influenced the decision to access and undergo radiation therapy [21, 22]. Henry et al. explored the reasons why patients in regional Victoria would not consider having radiation therapy. The authors found that duration of radiation therapy, proximity to radiation therapy services or alternatively access to transport and affordable accommodation was important. Disruption to work and family, and financial impact also influenced patient choices [23].

In the North American context, Billar et al. noted that radiation therapy utilisation stayed relatively stable when looking at approximately 180,000 patients over a 10-year period, primarily as whole breast radiation therapy utilisation had declined due to competing and more convenient treatment options like partial breast irradiation being utilised. While overall utilisation had not increased, the direct correlation to radiation therapy centre access was not explored [12]. Celaya et al. illustrated that distance and weather influenced patient’s decisions to have radiation therapy for breast cancer, suggesting in winter months’ patients were less likely to accept treatment [2]. Schootman et al. identified a similar trend for ductal carcinoma in situ [24]. Freeman et al. illustrated Appalachian women were more likely to opt for mastectomy [18] Wheeler et al. made the interesting observation that that patients living closer to a radiation therapy service were less likely to receive radiation therapy, and concluded that targeted rural outreach programs were effective in improving utilisation [25].

## Discussion

This review explores a number of themes. Baldwin et al. indicated a proportion of patients bypass their nearest centre, for reasons which are worth further exploration [11]. Clinical practice informs us this is probably a combination of complex factors including reputation, recommendation, familiarity with regions and location of family/relatives and job requirements. Patients may choose to have treatment where they are close to or where they can live with relatives to access - as can happen when siblings or children are in metropolitan areas. This provides psychological and practical support for the patient which enables treatment uptake.

Lengoc et al. raise the question of who are the enablers of regional and rural care, suggesting that family physicians/general practitioners play a crucial role in rural cancer care given their key role in the patient’s care [19]. This is a similar conclusion advocated by Jiwa et al. that indicated involvement of the general practitioner would result in better coordination of care, and better practical and psychosocial support [26]. This may be quite different to large metropolitan services, where the general practitioner plays a lesser role and it highlights that rural general practitioners are key stakeholders in rural cancer care.

Looking at ongoing drivers for establishing cancer centres, there is little in the literature. This is perhaps not surprising given that enablers and barriers from the point of view of health systems may not be explored and published in the academic literature but rather exist as issues discussed at a political level or in policy documents and may be confidential or in-house policy documents. This is an area worthy of further exploration as the need for de-centralised services around the world becomes essential to improve access to care in developed and developing countries.

Several studies explored patient decision-making to have or not to have radiation therapy although surprisingly few papers explored the reasoning for this. Women in rural areas are likely to travel greater distances to receive radiation therapy treatment and to stay away from home. They were as a result more likely to have a mastectomy and less likely to have radiation therapy after lumpectomy. The drivers for such decisions were generally social, economic or practical (work requirements, time off) rather than medical in nature. The literature also suggests that cost is a key driver; both the cost of health care itself and the loss of income. It is important to recognise that physicians play an influential role in the decisions that patients with breast cancer in rural areas make. Hence, ongoing professional development is important as physicians otherwise may lack knowledge about state-of-the art breast cancer treatments, thereby limiting the treatment options for rural women.

The literature suggested that health staff are the principal source of support for rural cancer patients. Once treatment is completed, cancer patients require ongoing additional support from health workers as well as their family members. One of the ways in which medical personnel can support cancer patients is by providing them with health, treatment-related and supportive services and information before, during and after treatment. These must be available through remotely accessible formats such as telephone (counselling), educational websites and informational mail sent out to cancer survivors. This will also be beneficial to enable information sharing among oncologists and other medical practitioners as well as helping keep rural general practitioners updated with the current and recommended treatments. Ultimately such efforts may result in better treatment compliance, satisfaction and health outcomes.

A number of studies included in this review explored the psychological needs of rural patients. Rural cancer patients have special needs because they experience feelings of isolation and disengagement which result in greater depressive symptoms, mostly due to a paucity of psychosocial support services. This review highlights that there are some constraints to receiving psychological services, particularly around issues of access. Moreover, rural patients may be less likely to seek mental health services due to negative attitudes held about mental health treatment and the challenges associated with anonymity in rural settings with a small population.

To determine a strategy that will improve radiation therapy utilisation and access, one needs to consider the information gap to be filled. The literature suggests we need to focus on research on understanding why patients in regional areas choose to or not to have radiation therapy at their nearest centre. This is probably best done by prospective qualitative approaches with patients and carers, to understand what social-economic and cultural factors are at play. Hypothesising that that many factors maybe specific to the region and people only. Doing this in partnership with government is probably a good strategy to reach knowledge translation aims rather than doing it retrospectively after radiation therapy centres have been established.

## Conclusions

To date, health system barriers and enablers to establishing a rural radiation therapy centre are poorly described in the academic literature. This review notes that resourcing of comprehensive oncology facilities remains lacking with the travel burden, distance and economic, on patients to their nearest facilities remains a barrier. This influenced where patients decided to undergo radiation therapy. Data indicates patient and health work awareness and knowledge around radiation therapy plays a role. These issues highlight the need for more comprehensive consideration of decision-making around the establishment of rural radiation therapy centres and the evaluation of their contributions. It is important to consider strategies to understand and correct perceptions and barriers that impact on policy decision making and that influence/inform patient preferences and decision making. There is a dearth of qualitative approaches that explore these issues.

## Abbreviations

PRISMA: Preferred Reporting Item for Systemic Reviews
